# Experimental Evaluation of the Mechanical Strengths and the Thermal Conductivity of GGBFS and Silica Fume Based Alkali-Activated Concrete

**DOI:** 10.3390/ma14247717

**Published:** 2021-12-14

**Authors:** Eliana Parcesepe, Rosa Francesca De Masi, Carmine Lima, Gerardo Maria Mauro, Giuseppe Maddaloni, Maria Rosaria Pecce

**Affiliations:** 1 Department of Engineering, University of Sannio, Piazza Roma, 21, 82100 Benevento, Italy; rfdemasi@unisannio.it (R.F.D.M.); germauro@unisannio.it (G.M.M.); giuseppe.maddaloni@unisannio.it (G.M.); 2 ITEMS s.r.l., Piazza Guerrazzi, 1, 82100 Benevento, Italy; c.lima@tesis-srl.eu; 3 Department of Structures for Engineering and Architecture, University of Naples Federico II, 80125 Naples, Italy; mariarosaria.pecce@unina.it

**Keywords:** alkali-activated concrete, slag, silica fume, experimental tests, mechanical properties, thermal properties, standard formulations

## Abstract

Alkali-activated concrete (AAC) could be a solution to use a cement-less binder and recycled materials for producing concrete reducing the carbon dioxide emission and the demand for raw materials, respectively. In addition to the environmental aspect, AACs can achieve mechanical characteristics higher than those of ordinary Portland concrete (OPC) but also an improvement of the thermal insulation capacity. Despite the positive results available in the scientific literature, the use of AACs in construction practice is still limited mainly due to the absence of codification for the mix design and consequently of specific design rules. In this paper, AAC produced by ground-granulated blast-furnace slag (GGBFS) and silica fume is investigated for the production of structural elements and to discuss the reliability of formulations for evaluating mechanical properties, necessary for structural design. The mechanical strengths (compression strength, tensile strength, flexural strength) are evaluated by experimental tests according to different curing times (7, 14, 28, 90 days) in ambient conditions and the thermal conductivity is measured to understand the effect that the material could have on thermal losses for a sustainable building perspective. The results showed that AAC strengths depend on the curing time and the exposure conditions, and the insulation properties can be improved compared to the traditional Portland cement with the proposed composition.

## 1. Introduction

The environmental and economic concerns associated with conventional cement-based building materials have led the scientific and technical community to explore possibilities in the use of alternative materials.

Currently, the concrete industry faces two main challenges that are the increasingly limited limestone reserves and the carbon taxes increase because of the growing demand for Portland cement (OPC) and the greenhouse gas emissions resulting from the production of OPC, respectively [1]. Indeed, approximately 5–7% of global CO_2_ emissions originate from the manufacturing of Portland cement [2,3], and its production consumes a high amount of energy that is accounting for 50–60% of the plant production costs [4].

These issues require the development of alternative binders, such as alkali-activated cement (AAC), with the aim of reducing the environmental impact of buildings, the use of a greater percentage of pozzolan waste and improving concrete performance [5]. From this point of view, they are considered an ecological binder whose production involves CO_2_ emissions lower than those necessary to produce OPC [6,7], with a reduction of greenhouse gas emissions up to 64% [8]. Other researchers found that the aluminosilicate material used for producing AAC such as fly ash and slag, which are both industrial by-products, have much lower carbon dioxide emission factors compared to cement: slag has been shown to release up to 80% less greenhouse gas emissions [9] and there are 80–90% less greenhouse gas emissions in the production of fly ash [10,11]. Based on a life cycle assessment (LCA), it was reported that the global warming potential of fly ash-based AAC is about 70% lower than that of OPC [12]. In addition, AAC is one of the alternative binders that attracts considerable attention also for its favorable engineering properties, that is, early compression strength, higher tensile strength, low permeability, good chemical resistance and fire-resistant behaviour [13].

Despite the advantages mentioned above, the use of alkali-activated materials in buildings and civil engineering, that is, as structural material, is limited by the lack of consistent guidelines for the mixture proportioning design [14]; furthermore, many mechanical properties have to be defined as strength in compression and tension, elastic modulus, bond and more, in general, the response of the reinforced elements. Due to the diversity of raw materials, additives and mixed proportions of AAC, current research is not yet able to give a consistent conclusion on the mechanical characteristics and influencing factors, but this variety suggests that this material could be locally adapted with the aim of achieving suitable engineering properties [15].

This study was a part of a research activity that was aimed to evaluate the performance of ground-granulated blast-furnace slag (GGBFS) and silica fume-based alkali-activated concrete for the application as structural elements (i.e., blocks, panels). During the research activity, many mixtures were developed and the most promising is the one discussed herein. The main scope of the paper was to discuss the reliability of formulations for evaluating mechanical properties necessary for structural design. The mixture was mainly designed to obtain good structural performance, but considering the current attention to the multifunctional use of new materials and the sustainability of the buildings, the thermal behaviour is worthy of investigation, especially for realizing blocks. It is known that the improvement of the thermophysical properties is usually matched with a reduction of the density and thus of the mechanical performance, therefore finding a mix design that provides a material with good structural and thermal performance is the current challenge. Hence, experimental tests carried out at the Laboratory of Materials and Structures (LAMAS) of the University of Sannio (Benevento, Italy) were performed to evaluate the main structural strengths, such as the compression strength, the splitting tensile strength and the flexural strength, according to different curing time; in addition, the characterisation of the material was completed with the tests for determining the thermal conductivity performed in the Insulating Material Thermal Analysis laboratory (IMATlab) of the University of Naples Federico II (Naples, Italy). In the available literature, very few researchers consider the combined effect of slag and silica fume on the mechanical and thermal parameters of alkali-activated concrete, instead, the evaluation of thermal behaviour is usually investigated for lightweight aggregate foamed geopolymer concrete whose insulation properties can be attributed to the porosity and voids of the microstructure.

The experimental evaluation of the mechanical strength allowed us to analyse the reliability between measured values and those prevised by standards provisions for OPC, which usually provide the relationship for tensile and flexural strength as a function of the compression one, pointing out the unsuitableness of the current codes in describing the AAC behaviour. Formulations developed by previous authors specifically for AAC were also considered.

## 2. State of the Art

### 2.1. Composition and Current Applications

Alkali-activated materials (AAMs) are formed by the reaction between alumino-silicates precursors with an alkaline chemical activator. These products are also named geopolymers (GPC), but even if chemically similar they are based on different aluminosilicate precursors. Indeed, geopolymers are viewed as a subset of AAM with the highest Al and lowest Ca concentrations [16]. In this paper, AAM was experimentally investigated, but a wider state of the art is provided considering previous works on geopolymers, since the purpose of this work is to study the mechanical and thermal aspect of the alkali-activated binders, thus the feasibility of the proposed mixture to realize structural elements.

Specifically, the typical precursors for the alkali-activated systems are cementitious materials such as steel and blast furnace slag (SG), or pozzolanic materials such as fly ash (FA), metakaolin (MK), silica fume (SF), rice husk ash (RHA). Various alkali activators can be used to activate the binder, such as potassium hydroxide (KOH), sodium hydroxide (NaOH), potassium silicate (K_2_SiO_3_), sodium silicate (Na_2_SiO_3_), sodium carbonate (Na_2_CO_3_) or a combination of these alkalis [16]. In addition, water and fine and coarse aggregates used in the production of OPC and other additives (i.e., plasticizer, fibres) can be added to the paste to reach the required concrete properties.

The various alumina silicates resources that can be used, affect the microstructures and chemical properties, although many physical properties of geopolymers prepared from various aluminosilicate sources may appear to be similar: FA, that is the most commonly used and widely tested resource, has a higher reactivity and more durability than MK based geopolymer, while SG based ones are considered to have higher early strength and greater acid resistance than MK and FA based systems. [15,17]. Provis and Deventer [18] summarised the properties of raw materials used in geopolymer manufacture.

Regarding the production and the spread of these materials, the scientific interest in the field of geopolymers is considerably increased since 2016 [19] becoming one of the hotspots of international research in recent years leading to the involvement of a larger number of geopolymer suppliers companies.

Despite this, GPC has not yet obtained international acceptance as a building material mainly because the production cost of geopolymer is not yet competitive. Mathew et al. [20] estimated that the cost of geopolymer based on coal ash and granulated blast furnace slag can be more than twice that of OPC-based concrete if the difference in the rate of transport is considered; otherwise, normalizing this effect, the cost difference between the two types of concrete is equal to 7%. However, the social cost that includes the advantages in terms of ambient temperature processing, low carbon dioxide emissions, environmental friendliness, carbon dioxide reduction targets and reutilisation of waste must be considered. Another study [8] on the lifecycle impacts of geopolymers in comparison to OPC revealed that the financial costs could be about 10–40% cheaper compared to conventional cement-based concrete, but those benefits are only realisable given the most appropriate source of feedstock and the least cost transportation.

Practical applications of geopolymer in the field of civil engineering can be found in the USA as a solution for repairing highways and airport runways thanks to the short setting time of geopolymer cement, and in Australia both for prefabricated elements and in situ castings [21]. However, for the spreading of the geopolymer market more investigations on long-term behaviour and durability are required [16] and data on the practicality of using geopolymer concrete as structural reinforced elements are needed to develop the design procedures [22].

### 2.2. Previous Research on Fresh and Hardened Properties

Research on the AAC has shown that the main parameters affecting the fresh and hardened properties include:

- Chemical composition and mineralogy of the precursor materials;
- Quantity, shape (solid, liquid) and type of alkaline activator;
- Si/Al ratio of precursor materials and quantity of available calcium sources such as Portland cement, blast furnace slag and lime;
- Total water/solids ratio (precursors + alkaline salts);
- Curing conditions.

It should be clarified that the exact contributions of these parameters to the strength are still not fully clear, consequently the various mixture design methods proposed in the literature are usually empirical or experimental [23].

Regarding the precursor materials, mixtures with fine particles activated with alkali exhibit greater workability and require less water due to the reduction of porosity and to the increase of the surface in the finer particles [24,25]. The concentration level and type of the activator play an important role in the mechanical and microstructural properties of geopolymers: increasing the activator concentration beyond an ideal concentration, may not only prevent the strength development of the geopolymer, but also lead to detrimental effects such as efflorescence and brittleness [26]. Furthermore, the molarity of the activator, the superplasticizer and the water content strongly influenced the workability of geopolymer concrete [27,28]. In particular, the addition of naphthalene and polycarboxylate super-plasticizing additives increases the workability [29,30].

The water to geopolymer solids ratio has been observed to have an inverse relationship with the compressive strength of concrete, instead, it has a direct relationship with the workability, similar to that observed between water/cement ratio and behaviour of OPC concrete [31].

Curing temperature has a significant effect on the microstructural and mechanical strength development of the geopolymer system. Generally, the geopolymerisation is accelerated at a higher temperature than the ambient one: it was observed the existence of an optimum curing temperature (60 °C) that allows the achievement of the best physical and mechanical properties [32].

Several mixes of geopolymer concrete based on low calcium FA activated by solutions of sodium silicates and sodium hydroxide were analysed by various authors [31,33,34,35,36,37,38,39]. From these studies it is noted that conditioned curing at a high temperature for at least 24 h is generally necessary to obtain mean values of compression strength greater than 50 MPa; therefore, only prefabricated elements can be made that require a large space with a conditioned environment. Furthermore, the modulus of elasticity has a scattered correlation with compression strength showing that for the same value of compression strength, the stiffness of the geopolymer concrete is significantly lower than that of ordinary concrete. Indeed, according to Duxson et al. [40], the Young modulus of geopolymer concrete is affected by microstructure based on speciation of the alkali silicate activating solutions, while for conventional concrete the Young modulus depend on the properties of the aggregate [41]. This is one difference between geopolymer and normal concrete that can lead to a different result; in the latter, Young modulus depends on the properties of the aggregate. However, the use of the appropriate aggregate type and content can improve the elastic modulus of geopolymer concrete can be improved up to 14.4% than that of the OPC concrete with the same compressive strength [42]. In addition, environmental conditions can also affect the development of elastic modulus, as for the compression strength: Pan et al. [43] reported that higher elastic modulus of geopolymer paste can be achieved after its being exposed to 300 °C, whereas the elastic modulus of OPC paste was almost unchanged.

In contrast to the case of elastic modulus, previous studies showed that the tensile strength of GPC was usually higher than that of the OPC concrete at the same compressive strength. In particular, the tensile strength of geopolymer concrete is affected by the type of raw materials: for instance, the decrease in the amount of additional water [44], the increase in percentage replacement of MK with RHA [45] or with FA [46] as the precursor material, higher concentration activator [47] led to higher tensile strength.

Similarly, to the tensile strength, geopolymer concretes exhibit higher flexural strength than ordinary ones [42,48], and it is related to the type and dosage of source materials [49,50]. Experimental results indicated that the diversity of binder materials is beneficial to the flexural strength development and adding a certain amount of OPC to FA seems to be a good way to increase the flexural strength [51]. Moreover, the alkaline activator concentration contributes to the increase in flexural strength, but excessive concentration can reduce the efficiency of geopolymerisation [52].

Several researchers are focused on the evaluation of the incidence of the geopolymeric process on other properties as thermal conductivity, sound absorption and durability. However, the main results regard the geopolymer foam concrete [53] for which it is obtained that the thermal conductivity is in a range of 0.15–0.48 W/m K [54]. It has been also demonstrated [55] that for the fly ash-based lightweight geopolymer concrete, the increase in dry density and fine aggregate contents resulted in higher compressive strength and thermal conductivity. Henon et al. [56] have demonstrated that the thermal conductivity can vary from 0.35 to 0.12 W/m K for pore volume fraction values in a geopolymer foam between 65 and 85%.

It is worthy of investigation the analysis of the thermal behaviour of not aerated geopolymer concrete that can also show high structural characteristics. More in general it has been obtained that for the metakaolin-derived Na, NaK, and K geopolymers, the thermal conductivity is closely linked with the specific heat, with little variation in thermal diffusivity observed in different conditions [57]. The sand particles incorporated in the geopolymeric matrix increase the thermal conductivity and decrease the specific heat of the resulting mortar structure [58]. Wongkeo et al. [59] considering a geopolymer synthesised by using fly ash, sodium hydroxide (NaOH) solution and sodium silicate (Na_2_SiO_3_) have found a maximum of compressive strengths at 1.7 MPa and the minimum thermal conductivity at 0.31 W/m K. Baran et al. [60] have indicated that addition of perlite could influence on the physical properties of geopolymer products. Moreover, Sukontasukkul et al. [61] have indicated that the incorporation of PCM aggregate could improve the thermal storage and the insulation level.

Pantongsuk et al. [62] have found that the porosity increases with higher hydrogen peroxide (H_2_O_2_) and decreases with increasing bagasse ash content. The increase in porosity resulted in a decrease in compressive strength but with a significant reduction in thermal conductivity from 0.3194–0.4519 to 0.1532–0.1857 W/m K with 1–1.5 wt% H_2_O_2_ addition and 10–20 wt% bagasse ash replacement.

## 3. Experimental Investigations

### 3.1. Mixture and Manufacturing Process

**Figure 1:**
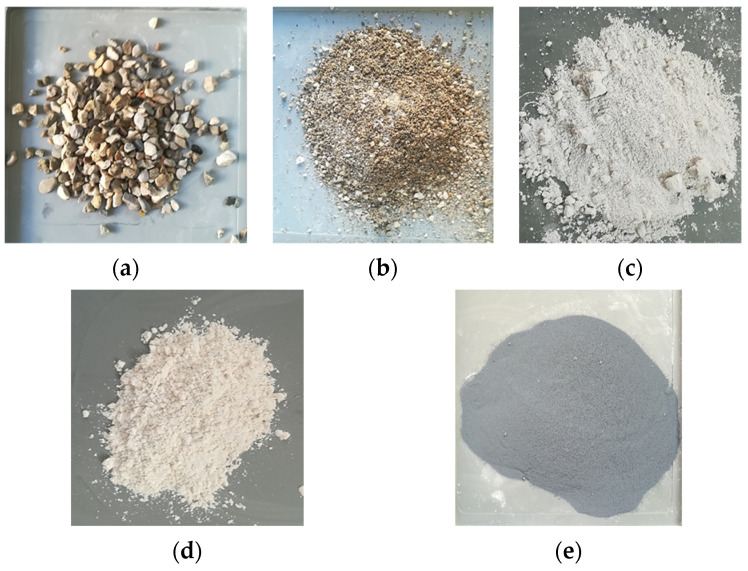
Solid constituents: (**a**) coarse aggregate, (**b**) sand, (**c**) limestone and gypsum, (**d**) slag, (**e**) silica fume.

The alkali-activated concrete used in these experimental tests included gravel (4–14 mm), sand (<4 mm), filler power with limestone and gypsum (<0.07 mm), grand granulated blast furnace slag (by-product of iron and steel-making) and silica fume (by-product of the silicon and ferrosilicon alloy production) as solid part (Figure 1). The chemical composition and properties of slag are summarised in Table 1.

The alkaline activator consisted of a commercially available sodium silicate (SS) solution having a modulus (R = SiO_2_/Na_2_O) of 1.6 with 27.50% by weight SiO_2_, 17.19% by weight Na_2_O, and a solid content of 44.69% by weight; water was used to complete the mix design. The addition of a naphthalene-based superplasticizer with a dosage of 2% by weight with respect to the binder improved its workability. Details of the mixture proportions used in this study are shown in Table 2. These proportions are the result of preliminary research activity, which was not part of the present work and included other partners, based on literature and tests with the aims of defining the best mixture in terms of both fresh and hardened properties.

Different researchers suggested several mixing methods based on the preparation of the liquid part one day before to make the polymerisation easier [35,63]. However, other authors have shown that the strength of the GPC is not significantly affected by the mixing process [64]; therefore, the mixing method applied in the present study does not need any pre-preparation resulting very similar to the process generally used for OPC concrete.

The mixing protocol applied in this study included the following steps:

- Step 1: all solids were mixed after quantifying by a mixer machine. The amount used was determined by the amount required for the number of specimens needed according to the mixture proportions reported in Table 2;
- Step 2: the liquids (alkali activator, superplasticiser and water) were poured over the solids and then they were mixed for about four minutes until a homogeneous compound was obtained;
- Step 3: the freshly prepared geopolymer concrete was poured into different type molds (cylindrical and prismatic depending on the test for which they were intended) in three layers and vibrated by means of a vibrating table to remove any entrapped air;
- Step 4: specimens were demolded within 48 h after casting and were stored in ambient conditions until the test day.

During the casting procedure, it was observed a fast setting of the mixture adhered strongly to the mold. The workability of AAC is generally lower than that of OPC concrete due to the presence of silicate in AAC would provide a sticky characteristic [64], therefore the use of a vibrating table is needed to compact well the geopolymer even for relatively low slump value, and the oiling of the molds is very important to guarantee a clean release of the samples. Furthermore, during the vibrating phase of the concrete, swelling of the mixture was observed, probably due to the heat release that occurs during the geopolymerisation process [65].

**Figure 2:**
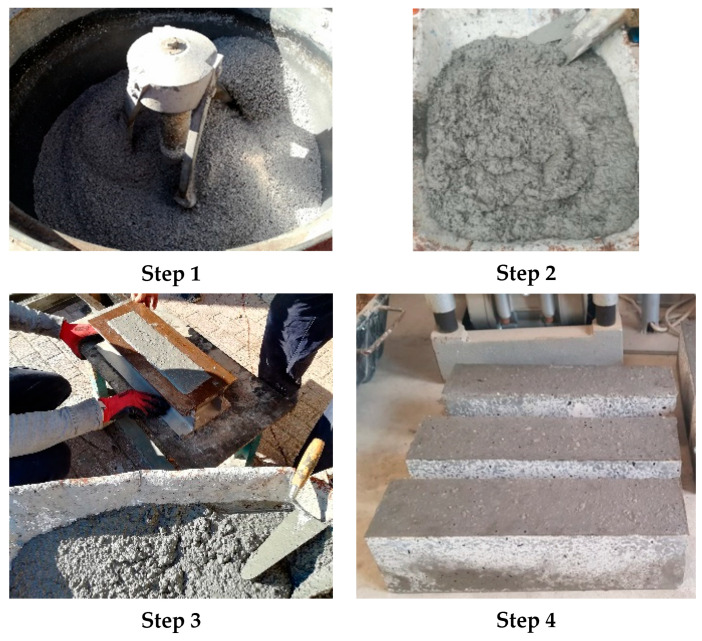
Alkali-activated concrete manufacturing process: **Step 1** mixing of the solids, **Step 2** homogeneous compound after the mixing of the liquids, **Step 3** molding and vibration of the specimens, **Step 4** storage of the specimens.

Hence, it is noted that the specimen’s preparation is a crucial phase that needs to be performed accurately for the correct success of the experimental program. Figure 2 shows the reportage of the step followed in the manufacturing.

### 3.2. Test Methods

The test program consists of mechanical and thermal characterisation. In Table 3 information about the testing procedure (standard references, measured properties, number and type of specimens) is summarised, while the details are presented in the following sections.

#### 3.2.1. Mechanical Tests

Mechanical characterisation was carried out according to the standards EN 12390-3 [66], EN 12390-6 [67], EN 12390-5 [68] for compression, tensile and flexural tests, respectively.

In particular, the compression tests were performed on cylinders with height-to-diameter ratio h/d = 2 applying a compressive axial load to molded cylinders until failure occurs. The cylindric compression strength *f_c_* of the specimens was determined as follows:

(1) $$f_{c}=F/A$$

where F is the maximum load attained during the test and A is the cross-sectional area of the concrete specimen.

As for the compression test, cylindrical specimens (h/d = 2) were used in the tensile splitting test. This test method applies a compressive load along the length of a cylinder until failure occurs. This loading causes tensile stresses in the plane containing the applied load, and relatively high compressive stresses in the area immediately around the applied load. Splitting tensile strength $f_{ct}$ was determined as follows:

(2) $$f_{ct}=\frac{2F}{\pi hd}$$

where F is the maximum applied load indicated by the testing machine, h and d are respectively the length and the diameter of the specimen.

Four-point bending tests were performed on 100 × 100 × 400 mm geopolymer beams. In this type of test vertical forces are applied to the beam in two points at 1/3 and 2/3 of the span length between the supports (S), allowing to achieve a constant bending moment without shear stresses in the length between the applied forces, thus ensuring pure bending failure. At failure, the flexural strength of the specimen $f_{cf}$ was calculated using the following formulation:

(3) $$f_{cf}=\frac{F\;\cdot \;S}{b\;\cdot \;d^{2}}$$

where F is the maximum load supported by the specimen, S = 300 mm is the span length between the supports, b = 100 mm and d = 100 mm respectively, the breadth and the depth of the specimen section.

All the specimens were cured in laboratory conditions and tested after 7, 14, 28, 90 days from casting for compression tests and after 28, 90 days both for tensile and flexural tests in order to evaluate the short- and long-term strength performance. Each mechanical test was conducted on three specimens per age, for a total of 24 specimens, therefore in the following section, the average results with their measure of statistical dispersion are reported according to the testing age.

#### 3.2.2. Thermal Tests

The Guarded Hot Plate (GHP) instrument is used for the measurement of the thermal conductivity (λ) of two specimens according to the standard ISO 8302 [69]. Several researchers are done with the application of the same principle for determining the conductivity of geopolymeric concrete. For instance, Lach et al. [70] have studied the thermal conductivity by varying the hydraulic additives; Kozub et al. [71] have found that the addition of glass wool waste can reduce the thermal conductivity coefficient and the amount of 5% by weight of glass wool waste causes a reduction of −6.6% in relation to the reference sample.

**Figure 3:**
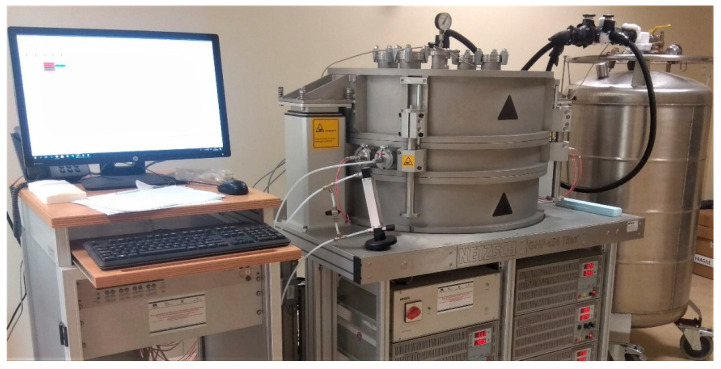
NETZSCH GHP 456 Titan^®^ system.

More in detail, The GHP principle is based on an absolute measurement method and therefore requires no calibration standards. The NETZSCH GHP 456 Titan^®^ system, high-temperature version [72] was used; the mean specimen temperature range is from −160 °C to 600 °C without the risk of thermally induced deformation and it requires liquid nitrogen for the sub-ambient temperature range. The plates are made of tungsten alloy with a dimension of 300 mm × 300 mm. The system can measure the thermal conductivity from 0.003 W/m K to 2 W/m K with an accuracy of 2% and reproducibility <1%. Figure 3 shows the NETZSCH GHP 456 Titan^®^ machine used in the above test.

The measurement is done by means of two plates with different temperatures and a guard ring that must host two samples of the material with the same thickness *t*. Thus, the samples are placed between two heated plates set to different temperatures. These temperatures are regulated by means of the heating (electrical power supply) and cooling (liquid nitrogen) systems. During the test, the desired temperature difference Δ*T* is established between the hot and the cold plates. The guard ring has the aim to minimize lateral heat losses. When the steady-state conditions are reached, the thermal conductivity is calculated according to the Fourier law, as reported in Equation (4) where the factor 2 is present because the measure is performed on two samples:

(4) $$\lambda =\frac{\dot{Q}\;\cdot \;t}{\Delta T\;\cdot \;2A}$$

herein:

- $\dot{Q}$ is the thermal power input to the hot plate, generated electrically thanks to the Joule effect;
- t is the average thickness of the sample, and Δ*T* is the temperature difference;
- A is the front area of the sample, equal to the hot plate area, i.e., 0.30 m × 0.30 m, otherwise filling materials (spacers) have to be used;
- the factor 2 is present because the measure is performed on two samples.

## 4. Results and Discussion

### 4.1. Mechanical Tests

The mass and the dimensions of the specimens were measured before conducting the tests in order to calculate the average unit weight of the geopolymer concrete that resulted in approximately 2300 kg/m^3^, which is slightly lower than the density of OPC concrete.

Table 4 presents the results of the experimental program: the results of each specimen are reported ($f_{i}$), then the mean value ($f_{m}$) of compression strength, tensile strength and flexural strength with the associated coefficient of variation (COV = standard deviation divided by mean value) are calculated according to the test age.

According to previous studies [63,73,74], the experimental results showed that most of the compression strength is gained already in the first 7 days from casting, but it is also noted that the curing time consistently affected the performance. Indeed, the value of *f_cm_* approximately doubled from tests performed at 7 days of curing to those at 14 days and improved from 7 days to the long-time, but a reduction of about 16% and 43% has been observed at 28 and 90 days compared to 14 days, respectively. Regarding the dispersion of the data, a higher value of COV (up to 23%) affected the measures at curing times greater than 14 days.

All the measured properties at 90 days showed a degradation of the strength; this effect could be related to the curing conditions in environments with high humidity or with uncontrolled humidity, as happened for the tested specimens, which played an important role in the development of compressive strength [75]. Indeed, when the products are exposed to humid air the phenomenon of efflorescence, and sub florescence, might occur due to the high alkalinity and the high mobility of alkalis, and the process of the loss of alkalis can affect the compressive strength. In particular, the phenomenon of sub fluorescence, which is not externally visible taking place under the surface of the material, leads to crystallisation pressure, which may exceed the tensile strength of hardened binders and generate structural damage [76,77]. The long-term strength loss was acknowledged also by Humad et al. [78] especially for sodium silicate–activated mixes, like the one analysed in this work, that showed a significant degree of carbonation with highly cracked areas already after 12 months of storage in a laboratory environment (20 °C ± 2 °C and 40% ± 7% RH). However, further experimental tests are necessary to investigate the long-term properties of the proposed mixture also with microscopic analysis, in order to have a complete characterisation of the material.

**Figure 4:**
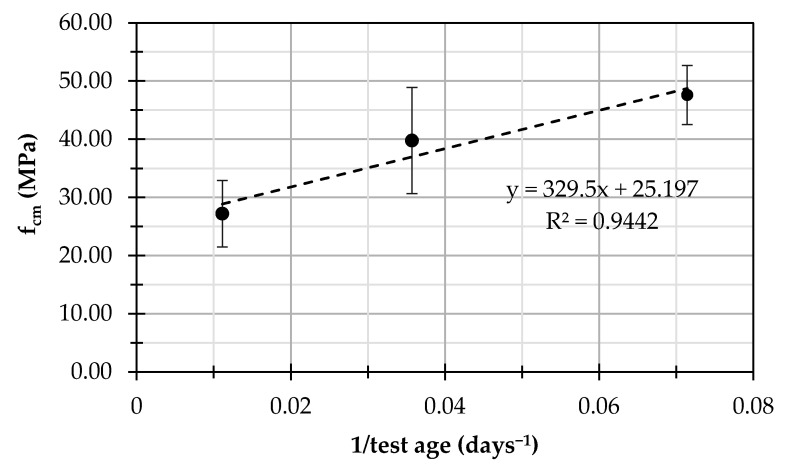
Linear regression of the compressive strength as a function of the inverse of the curing time.

As the reduction of compression strength was observed for curing times greater than 14 days, the experimental mean values of compression strength, also indicating the standard deviation for each experimental value, are plotted with reference to the inverse of the test age (Figure 4). This allows the identification of an asymptotic value of strength through a linear regression (with a coefficient of determination R^2^ = 0.94) that is equal to 25.2 MPa.

The behaviour of the AAC identified in this study does not reflect that of the ordinary concrete cured in standard conditions for which the increase in strength is expected over time. Therefore, the qualification rules in terms of control times and environmental conditions used for the ordinary concrete cannot be applied to this material, but new procedures need to be developed especially for the identification of the design strength. In particular, if standardised mixtures are defined a specific set of tests in compression at different curing times can be established (three ages at least) to extrapolate the effective resistance at a long time by the proposed procedure.

In addition, the reduction of the compressive strength is relatively more considerable than that of the tensile and flexural strength; the latter varies much lower compared to the tensile strength showing also lower dispersion of data.

However, further analyses by monitoring the microstructural and mineralogical developments are needed to better understand the behaviour of the material also for durability issues in the long term.

**Figure 5:**
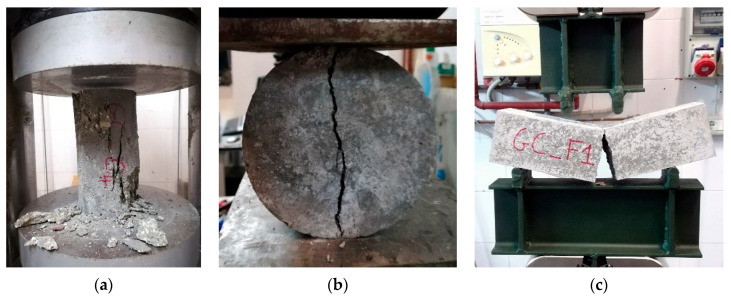
Failure of specimens: (**a**) compression test, (**b**) splitting tensile test, (**c**) flexural test.

Figure 5 shows the failure mode of the specimens typically obtained after the test.

In order to analyse the tensile and bending resistance of AAC, it is useful to compare the experimental results reported in Table 4 with those expected applying formulations proposed by codes for OPC or by technical literature specific for alkali-activated materials. In particular, the following standards and relationships for OPC are considered:

- Eurocode 2 (EN 1992) [79] for strength class of concrete C ≤ 50/60
  (5) $$f_{ctm}=0.30\;\cdot \;{\left(f_{ck}\right)}^{2/3}\;with\;f_{ck}=f_{cm}-8\;;\;\;\;\;\;\;\;\;\;f_{cfm}=1.2\;\cdot \;f_{ctm}\;\;\;\;\;\;\;\;\left(\mathrm{MPa}\right)$$
- American Standards (ACI 318) [80]
  (6) $$f_{ctm}=0.56\cdot {\left(f_{cm}\right)}^{1/2};\;\;\;f_{cfm}=0.62\;\cdot \;{\left(f_{cm}\right)}^{1/2}\;\;\;\;\left(\mathrm{MPa}\right)$$
  where $f_{ctm}$ and $f_{cfm}$ are the mean value of splitting tensile strength and flexural strength, respectively, and $f_{cm}$ is the mean value of cylinder compression strength.

Various studies have acknowledged that geopolymer concrete shows a better performance in tension compared to the provisions for OPC [35,48,49,81] especially for heat-cured geopolymer concrete [47]. This aspect has led several authors to make attempts for the development of new specific empirical correlations considering the experimental result of all curing conditions and ages, instead, the previous research lacks new formulations for the flexural behaviour. Ryu et al. [82] found that the splitting tensile strength ($f_{ct}$) with respect to the cylindric compressive strength ($f_{cm}$) is lower than that provided by the formulae of ACI 363R-92 [83] and Model Code [84], proposing hence the following Equation (7):

(7) $$f_{ct}=0.17{\left(f_{cm}\right)}^{3/4}\;\;\;\;\left(\mathrm{MPa}\right)$$

Other formulations based on experimental investigations were suggested by Lee-Lee [85] (Equation (8)), Hardjito [86] (Equation (9)), Nguyen et al. [35] (Equation (10)) for the splitting tensile strength $f_{ct}$ as a function of the compressive cylinder strength $f_{cm}$:

(8) $$f_{ct}=0.45{\left(f_{cm}\right)}^{1/2}\;\;\;\;\left(\mathrm{MPa}\right)$$

(9) $$f_{ct}=0.7{\left(f_{cm}\right)}^{1/4}\;\;\;\;\left(\mathrm{MPa}\right)$$

(10) $$f_{ct}=0.858{\left(f_{cm}\right)}^{0.41}\;\;\;\;\left(\mathrm{MPa}\right)$$

**Figure 6:**
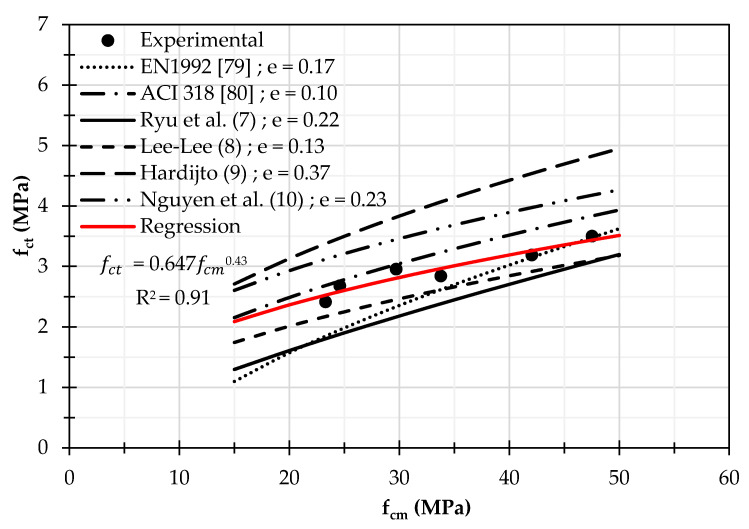
Comparison between the experimental values of tensile strength, the experimental relationships for AAC proposed in the literature, and the standard relationships for OPC depending on the compression strength.

The comparison between the experimental values of the splitting tensile strength and the aforementioned formulations is reported in Figure 6; the experimental results are used considering the tests at 28 and 90 days of curing. The mean error between the experimental and the theoretical values ($e=\sqrt{\left(\overset{N}{\underset{i}{\sum }}{\left(\left(f_{cti,exp}-f_{cti,th}\right)/f_{cti,exp}\right)}^{2}\right)/N}$ where *N* = 6 is the number of measured values, $f_{cti,exp}$ and $f_{cti,th}$ are the value of tensile strength measured from tests and calculated by codes, respectively) is calculated and reported also in Figure 6 for the various formulations.

The ACI formulation gives the best fitting, but it is unsafe, while quite the same fitting is given by Equation (8) that is also safe because the raw materials used and the curing conditions applied in this study are similar to those reported in Ref. [85]. Instead, the difference between the other relationships proposed by the previous authors is probably due to the variability of the aluminosilicate material used in the mixture composition; therefore, it would be important to identify more standardised mixtures and achieve a classification, also considering economic aspects, to be able to define reliable formulae of the mechanical characteristics.

Considering the experimental measures from Figure 6, the regression with the same approach of OPC is developed obtaining R^2^ = 0.91, which confirm the efficiency of the type of formulation.

**Figure 7:**
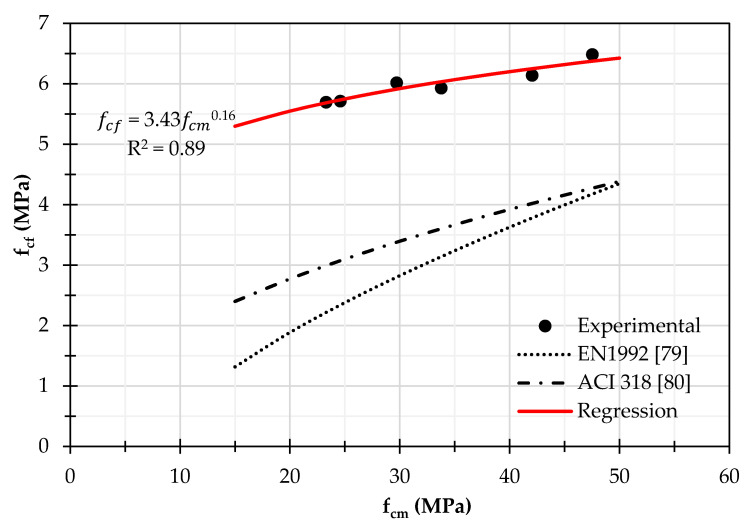
Comparison between the experimental values of flexural strength and the theoretical values calculated according to the standard for OPC.

Regarding the tensile resistance in flexure, Figure 7 shows the comparison between the experimental values and the theoretical ones calculated according to the standards Eurocode 2 and ACI 318 for OPC as a function of the $f_{cm}$; it results up to double of the standard provisions, that is also double of the resistance measured in pure tension. In Figure 7 the regression formulation of the experimental results is also reported in the form $f_{cf}=k{\left(f_{cm}\right)}^{\alpha }$, that seems the efficient approach for OPC. The improved flexural performance of about 60% of geopolymer concrete with respect to the ACI prediction was acknowledged also by Warhono et al. [87]. These results depend on the stronger connection between geopolymer binder and aggregate. Indeed, according to Lee & Deventer [88], the use of soluble silicates promotes greater interparticle bonding within the geopolymer binders to the aggregate surface than the case of ordinary concrete, therefore it is more difficult to cut the link between them. The higher cohesive behaviour does not improve the tensile resistance but increases the ultimate bending moment due to a post-peak contribution of the constitutive relationship in tension, that results in a higher resistance in flexure.

### 4.2. Thermal Tests

Thermal conductivity is the most important thermal property that affects heat transfer by conduction through concrete. The adoption, also for structural elements, of concrete with low thermal conductivity, contributes to reducing the heat losses of the building envelope and thus the building energy consumptions. Specifically, the thermal conductivity indicates the quantity of heat transmitted through a unit thickness in a direction perpendicular to a surface of the unit area, due to a unit temperature gradient under given conditions. It influences the conduction heat transfer. Two geopolymer panels have been used for the measurement of thermal conductivity. The operation temperature of such panels, considering typical ranges for the buildings, can generally vary between −10 °C and 50 °C in most climatic zones, worldwide. Therefore, this latter property is measured at three temperature (T) levels, i.e., −10 °C, 20 °C and 50 °C. In this regard, a temperature difference (Δ*T*) of 6 °C between the hot plate and cold ones is set in all cases. Table 5 shows the results.

First of all, it can be noted that the thermal behavior varies with the operative temperature since the thermal conductivity can increase by more than 25% from −10 °C to 50 °C. However, there is a regular slope of around 12% each 30 °C of temperature variation. A slight increase was expected but the magnitude of the increment is not comparable with other values in the literature because researchers usually give only the measurement with one operative condition or considering extreme ranges of variation (20–800 °C). For instance, Wang et al. [89] for the fly ash concrete have found that the thermal conductivity reduced from 1.69 to 0.95 W/m K with temperature increment from 20 °C to 550 °C. Briefly, when the temperature exceeds the conventional value of a moderate environment, the changing of λ-value is attributable to chemical and physical changes in the concrete structure that is heterogeneous and permeable. Kim et al. [90] have stated that the humidity specimen’s condition and aggregate volume fraction are the main effective factors on the thermal conductivity of concrete. considered the effect of seven factors on the thermal conductivity of cement paste, mortar and concrete. For this reason, the values in Table 5 seem to suggest that the thermal conductivity, as the compressive strength, is influenced by the moisture content and thus the phenomenon of efflorescence and sub florescence also affect the insulation property. Other investigations are needed for evaluating the incidence of this phenomenon on long-term and the aging effect. Indeed, the installation in a humid environment could compromise the effectiveness of the insulation or the application of particular protective products with a high degree of breathability should be designed.

**Figure 8:**
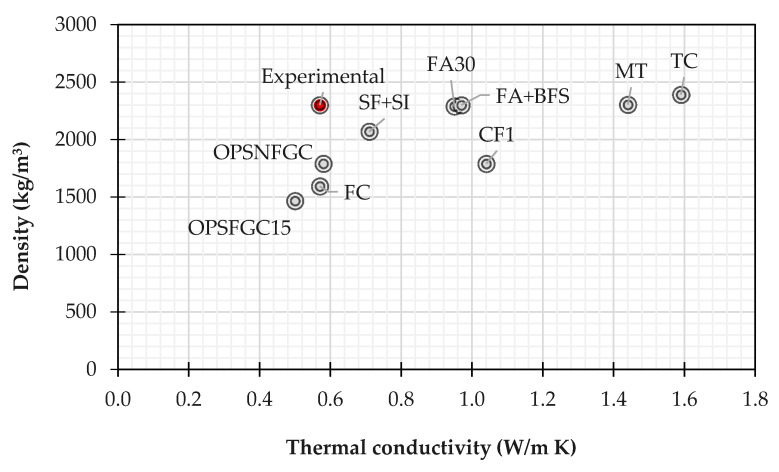
Comparison between measured thermal conductivity and available data from experiments in literature.

The value of measured thermal conductivity is influenced by the spatial spreading and volume ratio of mixed elements and voids created during the process also of a high-density material. For understanding the effect of adding slag and silica fume to the production process, the value measured at 20 °C is compared with the thermal conductivity of other geopolymer concrete (foamed or not) and traditional materials as reported in the scientific literature. Figure 8 proposes the comparison of thermal conductivity of the tested sample with other values measured and reported in the literature; herein:

- TC: traditional concrete with high density [91];
- MT: traditional mortar [91];
- CF1: modified concrete with fly ash (FA1) [91];
- OPSNFGC and OPSFGC15: oil palm shell respectively non-foamed and foamed geopolymer concrete [92] utilizing waste materials such as low-calcium fly and palm oil fuel ash as cementitious materials, and oil palm shell as lightweight coarse aggregate.
- FA30: concrete with fly ash in 30% cement replacement [93];
- FA+BSF: concrete with fly ash and blast furnace in 30% cement replacement [93];
- FC: foamed concrete with medium density [94];
- SF+SI: concrete containing 2% silane and 15% silica fume as admixture [95].

As seen from Figure 8, the experimental mixture exhibited lower thermal conductivity compared to the conventional materials (MT and TC) with comparable density. The results prove that the added materials give higher thermal resistivity compared to the conventional mixture without compromising the structural performance. The reduction in the conductivity is about −40% compared to the modified concrete with fly ash and blast furnace (FA30 and FA+BSF) also if the density is comparable thus the decrease in the thermal conductivity cannot be attributed to the increase of void ratio that should decrease the unit weight of concrete but to the property of slag and silica fume.

Moreover, it can be noted that the measured conductivity is comparable to non-foamed geopolymer concretes (lower than −2.0%) and to foamed concrete but the density is higher respectively of 28% and 44%. The improvement compared with the foamed concrete is not expected because in this case, the improvement of thermal conductivity is due to foaming agents that generate a large volume of artificial pores in hardened pastes, leading to increasing the thermal resistance. However, the foamed binder pastes are usually not suitable for structural purposes, due to their significantly low strength. Moreover, literature findings indicate that for reducing the thermal conductivity of concrete two solutions can be adopted, or the replacement of normal weight aggregates by lightweight aggregates or the replacement of Ordinary Portland Cement by supplementary cementing materials; however, these techniques generally compromise the mechanical properties as in the case of foamed concrete.

Thus, the purpose of the proposed experiment is to demonstrate that the obtained mixture conjugates good thermal and mechanical properties.

In addition to affecting the structural behavior, the higher value of density positively influences the thermal inertia/capacity enhancement of the building envelope, which is highly effective during the cooling season because it increases lag and attenuation of the heatwave.

Finally, it wants to be remarked that this comparison is affected by several factors that can modify the mix design of a specific mixture, and the final results can be largely get affected by such parameters. In the future, some other mixes will be prepared and utilised for comparison under the same thermal testing conditions.

## 5. Conclusions

In this research, the mechanical and thermal properties of alkali-activated concrete produced by silica fume and GGBFS were investigated by experimental tests. The main results are summarised below:

- A rapid setting of the mixture has been observed during the manufacturing process, therefore, the difficult workability can be a challenge to face during casting;
- The compression strength of alkali-activated concrete is affected by test age and significant performance is obtained already in the early days of curing;
- The long-term measured properties showed a degradation of the strength probably due to the curing conditions in humid environments that could favor the development of the sub fluorescence phenomenon and the carbonatation causing internal damage to the material. In addition, it is necessary to define new qualification procedures and identify more standard mixtures because AAC is sensitive to the curing regime. The proposal could be a procedure with a fixed number of tests for various curing ages and the extrapolation of the long-term resistance;
- The measured values of tensile strength $f_{ct}$ is in good agreement with the formulation proposed by ACI 318 for OPC, but it is unsafe, while quite the same good fitting is obtained by Lee-Lee formulation that is also safe. However, other formulations proposed in the literature for AAC and GPC do not give good fitting confirming the strong dependence of the mechanical properties from the mix design;
- The experimental flexural strength resulted much greater (quite double) than the values calculated applying the formulations of codes as a function of the compressive strength or strength in pure tension. This phenomenon is due to a higher interparticle bond between geopolymer binder and aggregate, but also in this case the result cannot be generalised for all the mix designs. Anyway, the relation of the tensile strength in flexure with the strength in compression is well fitted by a formula as $f_{cf}=k{\left(f_{cm}\right)}^{\alpha }$;
- The measured thermal conductivity is affected by the test temperature but the reference value at 20 °C is 0.57 W/m K. This value is about 60% lower than traditional products with the same density and 14% higher compared with foamed geopolymer concrete with half the density value. The adoption of structural and non-structural building components would help to reduce the heat losses (low thermal conductivity) but also the overheating problem during the summer period (thanks to high density) and thus the yearly energy consumption.

Finally, the results of the experimental campaign give preliminary information on the potential of the proposed mix for the application in the construction sector as bearing block or blocks for infill walls due to high compressive strength, good performance in tension and lower thermal conductivity. Additional experimental and simulation studies are needed both to complete the understanding from the mechanical side, such as in the fields of multiaxial stress states and long-term mechanical properties, both to evaluate the effect of different environmental conditions on the thermal conductivity and also to test the amount of energy savings. Nevertheless, a standardisation of the mix design supported by specific procedures of qualification (type, numbers and curing ages of the specimens) and design formulations are necessary to really employ the material.

## Figures and Tables

**Table 1:** Chemical composition and physical properties of GGBFS used.

Component/Property	Slag
FeO (%)	<1.3
CaO (%)	38–45
SiO_2 (%)_	32–37
Al_2_O_3_ (%)	13–16
MgO (%)	5–8
TiO_2_ (%)	<1.5
MnO (%)	<0.5
Available alkali	<0.5
SO_3_ (%)	2–3
S (%)	<1.0
Cl (%)	<250 ppm
Fineness (m^2^/kg)	440
Specific gravity	2.85
Bulk density (kg/m^3^)	1200

**Table 2:** Mixture proportions of experimental concrete.

Constituents	Mass (kg/m^3^)
Gravel (4–14 mm)	471
Sand (<4 mm)	1092
Limestone and gypsum (<0.07 mm)	128
GGBFS	224
Silica fume	48
Alkali activator	170
Superplasticizer	8
Water	140

**Table 3:** Summary of the tests.

Type	Reference	Details
Compression test	EN 12390-3 [66]	Type of specimens: cylinder h/d = 2 Number of specimens: 3 specimens per age Test age: 7, 14, 28, 90 days Measured properties: compression strength
Splitting tensile test	EN 12390-6 [67]	Type of specimens: cylinder h/d = 2 Number of specimens: 3 specimens per age Test age: 28, 90 days Measured properties: tensile strength
Four-point bending test	EN 12390-5 [68]	Type of specimens: beam 100 × 100 × 400 mm Number of specimens: 3 specimens per age Test age: 28, 90 days Measured properties: flexural strength
Thermal test	ISO 8302 [69]	Type of specimens: Block: 300 × 300 × 500 mm Number of specimens: 2 specimens of same age Test age: 90 days Measured properties: thermal conductivity

**Table 4:** Results of mechanical tests.

Property	Test Age (Days)	ID_specimen	$f_{i}\;(\mathrm{MPa})$	$f_{m}\;(\mathrm{MPa})$	COV (%)
Compression strength	7	GC_C1 GC_C2 GC_C3	23.21 21.29 28.70	24.40	16
	14	GC_C4 GC_C5 GC_C6	41.75 50.36 50.68	47.59	11
	28	GC_C7 GC_C8 GC_C9	42.04 47.51 29.71	39.75	23
	90	GC_C10 GC_C11 GC_C12	33.76 24.58 23.28	27.21	21
Tensile strength	28	GC_T1 GC_T1 GC_T3	3.52 3.19 2.96	3.22	9
	90	GC_T4 GC_T5 GC_T6	2.41 2.48 2.68	2.65	8
Flexural strength	28	GC_F1 GC_F2 GC_F3	6.02 6.49 6.14	6.22	4
	90	GC_F4 GC_F5 GC_F6	5.70 5.93 5.71	5.78	2

**Table 5:** Measurements of thermal conductivity.

λ (W/m K) at −10 °C	λ (W/m K) at 20 °C	λ (W/m K) at 50 °C
0.51 ± 0.01	0.57 ± 0.01	0.64 ± 0.01

## Data Availability

Data are contained within the article.

## References

[1] Singh B., Ishwarya G., Gupta M., Bhattacharyya S.K. (2015). Geopolymer concrete: A review of some recent development. Constr. Build. Mater..

[2] Lawrence C.D., Heweit P.C. (1998). The production of low-energy cements. Lea’s Chemistry of Cement and Concrete.

[3] Pal A. (2018). Developing low-clinker ternary blends for indian cement industry. J. Inst. Eng. Ser. A.

[4] Wang J., Dai Y., Gao L. (2009). Exergy analyses and parametric optimizations for different cogeneration power plants in cement industry. Appl. Energy.

[5] Shi C., Fernandez-Jiminez A., Palomo A. (2002). New cements for the 21st century: The pursuits of an alternative to Portland cement. Cem. Conc. Res..

[6] Khale D., Chaudhary R. (2007). Mechanism of geopolymerization and factors influencing its development. J. Mater. Sci..

[7] Davidovits J. Environmentally Driven Geopolymer Cement Applications. Proceedings of the Geopolymer 2002 Conference.

[8] McLellan B.C., Williams R.P., Lay J., van Riessen A., Corder G.D. (2011). Costs and carbon emissions for geopolymer pastes in comparison to ordinary Portland cement. J. Clean. Prod..

[9] Roy D.M., Idorn G.M. (1982). Hydration, structure, and properties of blast-furnace slag cements, mortars, and concrete. J. Am. Concr. Inst..

[10] Duxson P., Lukey G., van Deventer J.S.J. (2007). Physical evolution of Na-geopolymer derived from metakaolin up to 1000 °C. J. Mater. Sci..

[11] Van Deventer J.S.J., Provis J.L., Duxson P., Brice D.G. (2010). Chemical research and climate change as drivers in the commercial adoption of alkali activated materials. Waste Biomass Valorization.

[12] Weil M., Dombrowski K., Buchwald A. (2009). Life-Cycle Analysis of Geopolymers.

[13] Amer I., Kohail M., El-Feky M.S., Rashad A., Khalaf M.A. (2021). A review on alkali-activated slag concrete. Ain Shams Eng. J..

[14] Van Deventer J.S.J., Provis J.L., Duxson P. (2012). Technical and commercial progress in the adoption of geopolymer cement. Miner. Eng..

[15] Zhang P., Wang K., Li Q., Wang J., Ling Y. (2020). Fabrication and engineering properties of concretes based on geopolymers/alkali-activated binders—A review. J. Clean. Prod..

[16] Provis J.L., van Deventer J.S.J. (2014). Alkali Activated Materials.

[17] Li C., Sun H., Li L. (2010). A review: The comparison between alkali-activated slag (Si+Ca) and metakaolin (Si+Al) cements. Cem. Concr. Res..

[18] Provis J.L., van Deventer J.S.J. (2009). Geopolymers: Structures, Processing, Properties and Industrial Applications.

[19] Ma C.K., Awang A.Z., Omar W. (2018). Structural and material performance of geopolymer concrete: A review. Constr. Build. Mater..

[20] Mathew M.B.J., Sudhakar M.M., Natarajan D.C. (2013). Strength, economic and sustainability characteristics of coal ash–GGBS based geopolymer concrete. Int. J. Comput. Eng. Sci..

[21] Aldred J., Day J. Is geopolymer concrete a suitable alternative to traditional concrete?. Proceedings of the 37th Conference on Our World in Concrete & Structures.

[22] Mo K.H., Alengaram U.J., Jumaat M.Z. (2016). Structural performance of reinforced geopolymer concrete members: A review. Constr. Build. Mater..

[23] Li N., Shi C., Zhang Z., Wang H., Liu Y. (2019). A review on mixture design methods for geopolymer concrete. Compos. B Eng..

[24] Kiattikomol K., Jaturapitakkul C., Songpiriyakij S., Chutubtim S. (2001). A study of ground coarse fly ashes with different finenesses from various sources as pozzolanic materials. Cem. Concr. Compos..

[25] Chindaprasirt P., Chareerat T., Hatanaka S., Cao T. (2011). High-strength geopolymer using fine high-calcium fly ash. J. Mater. Civ. Eng..

[26] Wang Y., Liu X., Zhang W., Li Z., Zhang Y., Li Y., Ren Y. (2020). Effects of Si/Al ratio on the efflorescence and properties of fly ash based geopolymer. J. Clean. Prod..

[27] Montes C., Zand D., Alouche E.N. (2012). Rheological behavior of fly ash-based geopolymers with the addition of superplasticizers. J. Sustain. Cem. Based Mater..

[28] Laskar A.I., Bhattacharjee R. (2011). Rheology of fly-ash-based geopolymer concrete. ACI Mater. J..

[29] Nematollahi B., Sanjayan J. (2014). Effect of different superplasticizers and activator combinations on workability and strength of fly ash based geopolymer. Mater. Des..

[30] Jang J.G., Lee N.K., Lee H.K. (2014). Fresh and hardened properties of alkali-activated fly ash/slag pastes with superplasticizers. Constr. Build. Mater..

[31] Hardjito D., Wallah S.E., Sumajouw D.M.J., Rangan B.V. (2004). On the development of fly ash-based geopolymer concrete. ACI Mater. J..

[32] Muñiz-Villarreal M.S., Manzano-Ramírez A., Sampieri-Bulbarela S., Gasca-Tirado J.R., Reyes-Araiza J.L., Rubio-Ávalos J.C., Pérez-Bueno J.J., Apatiga L.M., Zaldivar-Cadena A., Amigó-Borrás V. (2011). The effect of temperature on the geopolymerization process of a metakaolin-based geopolymer. Mater. Lett..

[33] Pan Z., Sanjayan J.G., Rangan B.V. (2011). Fracture properties of geopolymer paste and concrete. Mag. Concr. Res..

[34] Gunasekera C., Setunge S., Law D.W. (2017). Correlations between mechanical properties of low-calcium fly ash geopolymer concretes. J. Mater. Civ. Eng..

[35] Nguyen K.T., Ahn N., Le T.A., Lee K. (2016). Theoretical and experimental study on mechanical properties and flexural strength of fly ash-geopolymer concrete. Constr. Build. Mater..

[36] Thomas R.J., Peethamparan S. (2015). Alkali-activated concrete: Engineering properties and stress–strain behavior. Constr. Build. Mater..

[37] Fernandez-Jiminez A.M., Palomo A., Lopez-Hombrados C. (2006). Engineering properties of alkali-activated fly ash concrete. ACI Mater. J..

[38] Shehab H.K., Eisa A.S., Wahba A.M. (2016). Mechanical properties of fly ash based geopolymer concrete with full and partial cement replacement. Constr. Build. Mater..

[39] Sofi M., Van Deventer J.S.J., Mendis P.A., Lukey G.C. (2007). Engineering properties of inorganic polymer concretes. Cem. Concr. Res..

[40] Duxson P., Provis J.L., Lukey G.C., Mallicoat S.W., Kriven W.M., Deventer J.S.J.V. (2005). Understanding the relationship between geopolymer composition, microstructure and mechanical properties. Colloids Surf. A Physicochem. Eng. Asp..

[41] Neville A.M. (2000). Properties of Concrete.

[42] Joseph B., Mathew G. (2012). Influence of aggregate content on the behavior of fly ash based geopolymer concrete. Sci. Iran.

[43] Pan Z., Sanjayan J.G., Collins F. (2014). Effect of transient creep on compressive strength of geopolymer concrete for elevated temperature exposure. Cem. Concr. Res..

[44] Aliabdo A.A., Elmoaty A.E.M.A., Salem H.A. (2016). Effect of water addition, plasticizer and alkaline solution constitution on fly ash based geopolymer concrete performance. Constr. Build. Mater..

[45] Billong N., Kinuthia J., Oti J., Melo U.C. (2018). Performance of sodium silicate free geopolymers from metakaolin (MK) and Rice Husk Ash (RHA): Effect on tensile strength and microstructure. Constr. Build. Mater..

[46] Nuaklong P., Sata V., Chindaprasirt P. (2018). Properties of metakaolin-high calcium fly ash geopolymer concrete containing recycled aggregate from crushed concrete specimens. Constr. Build. Mater..

[47] Deb P.S., Nath P., Sarker P.K. (2014). The effects of ground granulated blast-furnace slag blending with fly ash and activator content on the workability and strength properties of geopolymer concrete cured at ambient temperature. Mater. Des..

[48] Ganesan N., Abraham R., Raj S.D., Sasi D. (2014). Stress-strain behaviour of confined geopolymer concrete. Constr. Build. Mater..

[49] Xie T., Ozbakkaloglu T. (2015). Behavior of low-calcium fly and bottom ash-based geopolymer concrete cured at ambient temperature. Ceram. Int..

[50] Altwair N.M., Johari M.A.M., Hashim S.F.S. (2012). Flexural performance of green engineered cementitious composites containing high volume of palm oil fuel ash. Constr. Build. Mater..

[51] Nath P., Sarker P.K. (2017). Flexural strength and elastic modulus of ambient-cured blended low-calcium fly ash geopolymer concrete. Constr. Build. Mater..

[52] Hamidi R.M., Man Z., Azizli K.A. (2016). Concentration of NaOH and the effect on the properties of fly ash based geopolymer. Procedia Eng..

[53] Dhasindrakrishna K., Pasupathy K., Ramakrishnan S., Sanjayan H. (2021). Progress, current thinking and challenges in geopolymer foam concrete technology. Cem. Concr. Compos..

[54] Zhang Z., Provis J.L., Reid A., Wang H. (2015). Mechanical, thermal insulation, thermal resistance and acoustic absorption properties of geopolymer foam concrete. Cem. Concr. Compos..

[55] Wang Y., Zheng T., Zheng X., Liu Y., Darkw J., Zhou G. (2020). Thermo-mechanical and moisture absorption properties of fly ash-based lightweight geopolymer concrete reinforced by polypropylene fibers. Constr. Build. Mater..

[56] Henon J., Alzina A., Absi J., Smith D.S., Rossignol S. (2015). Analytical estimation of skeleton thermal conductivity of a geopolymer foam from thermal conductivity measurements. Eur. Phys. J. Spec. Top..

[57] Duxson P., Lukey G.C., Van Deventer J.S.J. (2006). Thermal conductivity of metakaolin geopolymers used as a first approximation for determining gel interconnectivity. Ind. Eng. Chem. Res..

[58] Xu Y., Chung D. (2000). Effect of sand addition on the specific heat and thermal conductivity of cement. Cem. Concr. Res..

[59] Wongkeo W., Seekaew S., Kaewrahan O. (2019). Properties of high calcium fly ash geopolymer lightweight concrete. Mater. Today Proc..

[60] Baran P., Nazarko M., Włosińska E., Kanciruk A., Zarębska K. (2021). Synthesis of geopolymers derived from fly ash with an addition of perlite. J. Clean. Prod..

[61] Sukontasukkul P., Nontiyutsirikul N., Songpiriyakij S., Sakai K., Chindaprasirt P. (2016). Use of phase change material to improve thermal properties of lightweight geopolymer panel. Mater. Struct..

[62] Pantongsuk T., Kittisayarm P., Muenglue N., Benjawan S., Thavorniti P., Tippayasam C., Nilpairach S., Heness G., Chaysuwan D. (2021). Effect of hydrogen peroxide and bagasse ash additions on thermal conductivity and thermal resistance of geopolymer foams. Mater. Today Commun..

[63] Rangan B. (2010). Design and manufacture of fly-ash based geopolymer concrete. Concr. Aust..

[64] Talha Junaid M., Kayali O., Khennane A., Black J. (2015). A mix design procedure for low calcium alkali activated fly ash-based concretes. Constr. Build. Mater..

[65] Zhang Z., Wang H., Provis J.L., Bullen F., Reid A., Zhu Y. (2012). Quantitative kinetic and structural analysis of geopolymers. Part 1. The activation of metakaolin with sodium hydroxide. Thermochim. Acta.

[66] (2019). EN 12390-3:2019 Testing Hardened Concrete—Part 3: Compressive Strength of Test Specimens.

[67] (2019). EN 12390-5:2019 Testing Hardened Concrete—Part 5: Flexural Strength of Test Specimens.

[68] (2019). EN 12390-6:2019 Testing Hardened Concrete—Part 6: Tensile Splitting Strength of Test Specimens.

[69] ISO 8302:1991 Thermal Insulation (1991). Determination of Steady State Thermal Resistance and Related Properties. Guarded Hot Plate Apparatus.

[70] Łach M., Pławecka K., Bak A., Lichocka K., Korniejenko K., Cheng A., Lin W.-T. (2021). Determination of the influence of hydraulic additives on the foaming process and stability of the produced geopolymer. Foams Mater..

[71] Kozub B., Bazan P., Gailitis R., Korniejenko K., Mierzwinski D. (2021). Foamed geopolymer composites with the addition of glass wool waste. Materials.

[72] NETZSCH GHP 456 Titan. https://www.netzsch-thermal-analysis.com/en/products-solutions/thermal-diffusivity-conductivity/ghp-456-titan.

[73] Cheng H., Lin K.L., Cui R., Hwang C.L., Chang Y.M., Cheng T.W. (2015). The effects of SiO2/Na2O molar ratio on the characteristics of alkali-activated waste catalyst–metakaolin based geopolymers. Constr. Build. Mater..

[74] Wallah S.E., Rangan B.V. (2006). Low-Calcium Fly Ash-Based Geopolymer Concrete: Longterm Properties.

[75] Yousefi Oderji S., Chen B., Jaffar S.T.A. (2017). Effects of relative humidity on the properties of fly ash-based geopolymers. Constr. Build. Mater..

[76] Zhang Z., Provis J.L., Ma X., Reid A., Wang H. (2018). Efflorescence and subflorescence induced microstructural and mechanical evolution in fly ash-based geopolymers. Cem. Concr. Compos..

[77] Allahverdi A. (2015). Methods to control efflorescence in alkali-activated cement-based materials. Handbook of Alkali-Activated Cements, Mortars and Concretes.

[78] Humad A.M., Provis J.L., Habermehl-Cwirzen K., Rajczakowska M., Cwirzen A. (2021). Creep and long-term properties of alkali-activated swedish-slag concrete. J. Mater. Civ. Eng..

[79] (2005). EN 1992-1-1 Eurocode 2: Design of Concrete Structures—Part 1-1: General Rules and Rules for Buildings.

[80] American Concrete Institute (2014). ACI 318-14 Building Code Requirements for Structural Concrete.

[81] Sarker P.K. (2011). Bond strength of reinforcing steel embedded in fly ash-based geopolymer concrete. Mater. Struct..

[82] Ryu G.S., Lee Y.B., Koh K.T., Chung Y.S. (2013). The mechanical properties of fly ash-based geopolymer concrete with alkaline activators. Constr. Build. Mater..

[83] American Concrete Institute (1992). ACI 363R-92 State-of-the-Art Report on High-Strength Concrete.

[84] (1993). CEB-FIP Model Code 1990: Design Code.

[85] Lee N.K., Lee H.K. (2013). Setting and mechanical properties of alkali-activated fly ash/slag concrete manufactured at room temperature. Constr. Build. Mater..

[86] Hardjito D., Rangan B.V. (2005). Development and Properties of Low-Calcium Fly Ash Based Geopolymer Concrete. Research Report GC-1 2005.

[87] Wardhono A., Law D.W., Molyneaux T.C.K. (2016). Flexural strength of low calcium class f fly ash-based geopolymer concrete in long term performance. Mater. Sci. Forum.

[88] Lee W.K.W., Deventer J.S.J.V. (2004). The interface between natural siliceous aggregates and geopolymers. Cem. Concr. Res..

[89] Wang W., Lu C., Li Y., Li Q. (2017). An investigation on thermal conductivity of fly ash concrete after elevated temperature exposure. Constr. Build. Mater..

[90] Kim K.H., Jeon S.E., Kim J.K., Yang S. (2003). An experimental study on thermal conductivity of concrete. Cem. Concr. Res..

[91] Remesar J.C., Simon F., Vera S., Lopez M. (2020). Improved balance between compressive strength and thermal conductivity of insulating and structural lightweight concretes for low rise construction. Constr. Build. Mater..

[92] Jing Liu M.Y., Alengaram U.J., Jumaat M.Z., Mo K.H. (2014). Evaluation of thermal conductivity, mechanical and transport properties of lightweight aggregate foamed geopolymer concrete. Energy Build..

[93] Demirboğ R. (2007). Thermal conductivity and compressive strength of concrete incorporation with mineral admixtures. Build. Environ..

[94] Alengaram U.J., Al Muhit B.A., Jumaat M.Z., Jing M.L.Y. (2013). A comparison of the thermal conductivity of oil palm shell foamed concrete with conventional materials. Mater. Des..

[95] Xu Y., Chung D. (2000). Cement of high specific heat and high thermal conductivity, obtained by using silane and silica fume as admixtures. Cem. Concr. Res..

